# An apocrine mechanism delivers a fully immunocompetent exocrine secretion

**DOI:** 10.1038/s41598-021-95309-8

**Published:** 2021-08-05

**Authors:** Denisa Beňová-Liszeková, Lucia Mentelová, Klaudia Babišová, Milan Beňo, Tibor Pechan, Bruce A. Chase, Robert Farkaš

**Affiliations:** 1 Laboratory of Developmental Genetics, Institute of Experimental Endocrinology, Biomedical Research Center, Slovak Academy of Sciences, Dúbravská cesta 9, 84505 Bratislava, Slovakia; 2 Department of Genetics, Comenius University, Mlynská dolina, B-1, 84215 Bratislava, Slovakia; 3 Institute for Genomics, Biocomputing and Biotechnology, Mississippi State University, 2 Research Boulevard, Starkville, MS 39762 USA; 4 Department of Biology, University of Nebraska at Omaha, 6001 Dodge Street, Omaha, NE 68182-0040 USA

**Keywords:** Cell biology, Cell signalling

## Abstract

Apocrine secretion is a recently discovered widespread non-canonical and non-vesicular secretory mechanism whose regulation and purpose is only partly defined. Here, we demonstrate that apocrine secretion in the prepupal salivary glands (SGs) of *Drosophila* provides the sole source of immune-competent and defense-response proteins to the exuvial fluid that lies between the metamorphosing pupae and its pupal case. Genetic ablation of its delivery from the prepupal SGs to the exuvial fluid decreases the survival of pupae to microbial challenges, and the isolated apocrine secretion has strong antimicrobial effects in “agar-plate” tests. Thus, apocrine secretion provides an essential first line of defense against exogenously born infection and represents a highly specialized cellular mechanism for delivering components of innate immunity at the interface between an organism and its external environment.

## Introduction

Secretory tissues have been known for centuries—the Harderian gland was described in red deer in 1694^1^, human sweat glands were described by Purkinje in 1833^2^, and the three major distinct secretory mechanisms (merocrine, apocrine and holocrine) were delineated by Schiefferdecker in 1922^3^. Apocrine secretion remains the least well understood process, both in terms of its mechanism and purpose. We recently demonstrated that the salivary glands (SGs) of the holometabolous insect *Drosophila* use a non-vesicular transport and non-canonical secretory pathway to produce a massive apocrine secretion, and applied molecular genetic tools available in *Drosophila* to obtain insights into its underlying mechanism^4^.

The SGs are well known for their use of exocytosis (merocrine secretion) during pupariation to release a salivary glue secretion (Sgs), a mixture of eight proteins that affixes a newly formed puparium to a substrate^5^. Exocytosis^6–8^ is a well-defined process where vesicles formed in the *trans*-Golgi are translocated to targeted sites on the plasma membrane. After they undergo nucleation, zippering, budding, and priming, the coalescence of vesicles and membrane fusion leads to the release of the Sgs-glue into the extracellular space.

Unlike exocytosis, apocrine secretion releases entire pieces of the cell and does not require homotypic membrane fusion. Instead, apical protrusions generate cytoplasmic fragments inside a secretory lumen. Apocrine secretion is initiated about 16 h after the exocytotic release of the Sgs-glue in still-metabolically active SGs. Confocal and transmission-electron micrography demonstrate that during its most intense phase, apocrine secretion at the apical pole of SG cells releases large cell fragments and entire organelles, including mitochondria, fragments of the ER and Golgi apparatus directly into the lumen of the SGs^9^. Proteomic analyses revealed that the secretion comprises thousands of microsomal, mitochondrial, ribosomal, membranous, cytoskeletal, and even nuclear and nucleolar proteins. Strikingly, the nuclear DNA itself remains intact, even though many nuclear proteins are released^9^. Its purpose in the SGs and in general has remained an enigma.

Here we demonstrate that it provides innate immune components into the exuvial fluid that lies between the pupa and its protective case, thereby providing the first non-structural line of antimicrobial defense. This result redefines the role of SGs during insect metamorphosis: not only do SGs first produce the glue to affix the pupa to a substrate, they later provide the pupa with exuvial fluid that functions as an essential protective barrier. This result also offers a general insight into the function of apocrine secretion in facilitating innate immunity. Though materials secreted by an apocrine mechanism are inherently complex, some of the protein components from apocrine glands of evolutionary distant species appear to be identical^4,10^.

## Results

**Figure 1:**
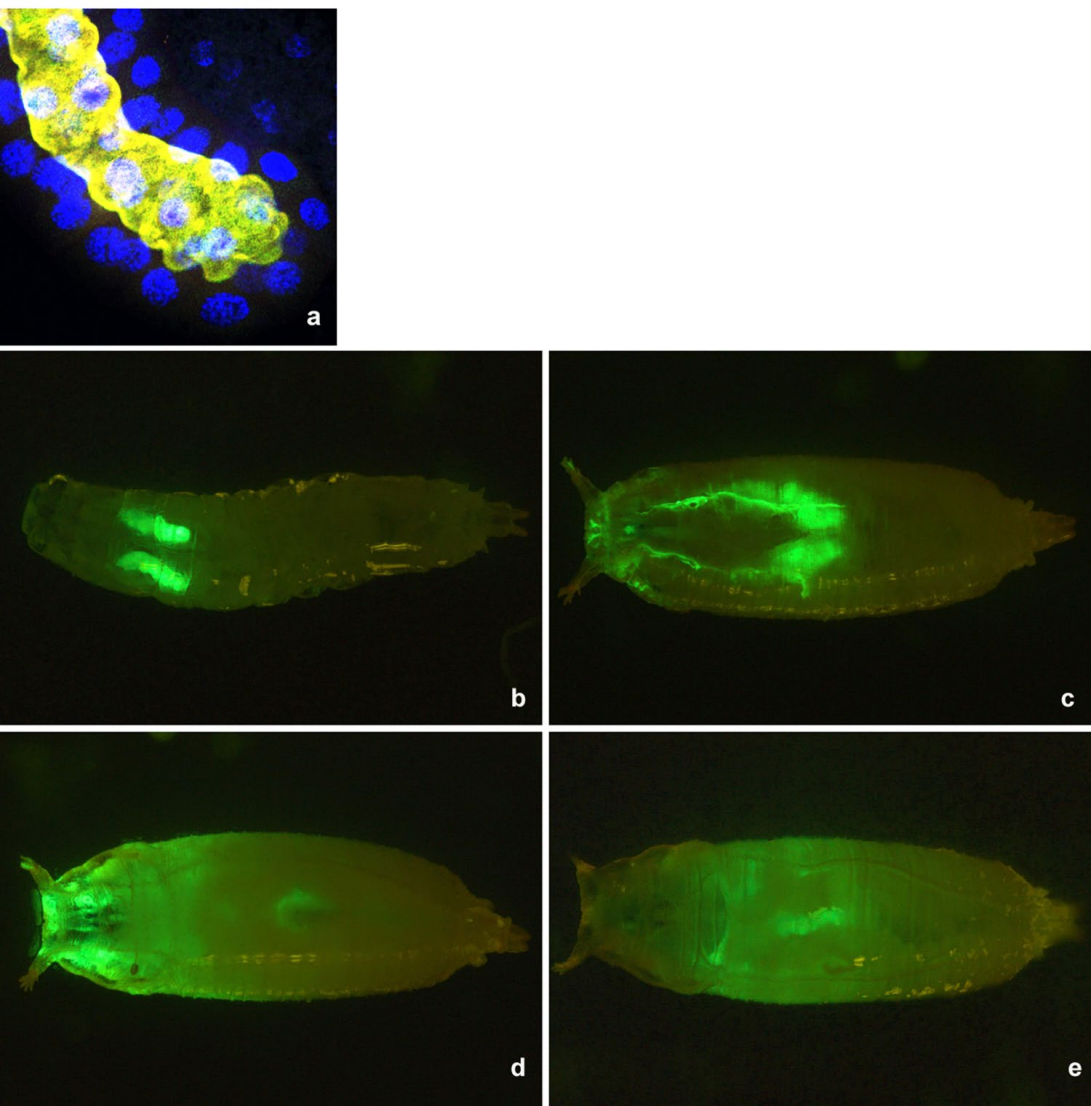
(**a**) Laser confocal view of the prepupal SG from an 8–10 h old wild-type prepupa having an apocrine secretion in its lumen. Visualized are tumor suppressor protein p127^l(2)gl^ (green), nuclear transcription factor BR–C (red), filamentous actin (dark blue), and DNA (light blue, Hoechst 33,258). The light yellow signal inside the lumen reflects the merging of the three proteinaceous signals. (**b**) Live imaging of late 3rd instar *Sgs3*-*GFP* larva. A strong GFP signal is found exclusively within the secretory granules inside the SGs. (**c**) When all of the Sgs-glue is not released from the SGs during pupariation, GFP signal is released into the SG lumen during apocrine secretion in the late prepupa at 8–10 h APF. (**d**) In the 12–13 h old prepupa (APF) the GFP signal becomes visible around the forming head capsula in the periexuvial space. (**e**) At the 5-h old pupa at 18 h APF, a more diffuse GFP signal is spread over almost the entire pupal body, concentrated inside the periexuvial space.

To address what purpose apocrine secretion could serve in the *Drosophila* SGs after glue secretion, we first traced the fate of proteins it releases into the SG lumen (Fig. 1a). Anatomical examination revealed that in the late prepupa, the pair of SGs share a single common salivary duct that only connects to the floor of the pharynx. This connection delivers the apocrine secretion into the periexuvial cavity instead of the alimentary tract, which becomes histolyzed shortly after pupariation. To verify release of the apocrine secretion into this space, we followed the fate of a GFP-marker strongly expressed in the late larval and prepupal salivary glands, *Sgs3-GFP* (Fig. 1b)^11^. Previously, we demonstrated that when Sgs3-GFP is strongly expressed in the late larval and prepupal SGs, the apocrine secretion exhibits GFP fluorescence and contains peptides corresponding to Sgs3 and GFP^9^. This is because the Sgs3-GFP fusion protein is incompletely released within the Sgs-glue prior to pupariation and remains stably present in the prepupal SGs. Therefore, we selected animals with high GFP fluorescence levels and followed the fate of the fluorescent signal during and following apocrine secretion. Signal released during apocrine secretion (at 8–10 h after puparium formation (APF)) enters the SG lumen (Fig. 1c) before being released during pupation (13 h APF) into the periexuvial space surrounding the head (Fig. 1d). Over the next several hours (15–22 h APF), it becomes almost evenly distributed over the pupa’s surface (Fig. 1e). The signal was detectable only in the space between the old and the newly formed pupal cuticle—the space that becomes filled with the exuvial fluid.

To verify GFP-Sgs3’s presence in the exuvial fluid, we isolated this fluid and characterized its proteins by MALDI-TOF/TOF proteomic analysis (M&M, Supplemental File). Several peptides corresponding to GFP were present at high coverage. We then combined MALDI-TOF/TOF and ESI–MS/MS approaches to undertake a detailed proteomic analyses on the apocrine secretion collected from the prepupal SG lumen and the pupal exuvial fluid. While many of its proteins were reported in our earlier study^9^, this more sensitive combination identified numerous additional proteins, including a large constellation of proteins used in innate immunity (Supplemental Table S1). The set of defense and immune-response proteins includes numerous antimicrobial proteins: cecropin A1, drosomycin, drosomycin-like 6, attacin, serpins, cathepsins, and immune-regulated catalase, functionally related components including various peptidases and peptidase inhibitors, and defense-response enzymes such as glutathione transferase. Interestingly, the proteins of the apocrine secretion and in exuvial fluid from 5- to 7-h old pupae (i.e., 18–20-h AFP, several hours after apocrine secretion) are nearly identical (Supplemental Table S1). This provides direct evidence that a major function of the SG apocrine secretion is to deliver, as components of a goulash containing many other proteins and non-proteinaceous components, immune-competent and defense-response proteins directly to the site where their action will be required during metamorphosis. Comparison of the SG-apocrine-secretion proteome to that of secretions from human apocrine glands (sweat, mammary, cerumenal, lacrimal, etc.)^4,12,13^ reveals that the major ontological categories of proteins are highly conserved and that all of these apocrine secretions share components of an innate immune response. Therefore, apocrine secretion appears to be an evolutionarily conserved mechanism to deliver innate immune components ready to implement an immediate defense response.

The mass spectrometric data also revealed that the SG apocrine secretion/exuvial fluid contains chitinase, Idgf3 and Idgf4-like chitinases, matrix metalloproteinase 1, dipeptidyl aminopeptidase III and chitin-binding proteins (ghost, dumpy), enolase, mannose-1-phosphate guanyltransferase, ubiquitin-specific proteases, and cysteine proteinases. These are all crucial components of exuvial fluid required for the digestion of the larval cuticle to facilitate successful molting. Earlier studies of the exuvial fluid from moths, beetles, and cockroaches, and even nematodes also identified proteins important for both molting and melanization, which occurs as an immunity response, with some demonstrating that molting fluids can inhibit the growth of bacteria in vivo^14–20^. While some of these studies are not directly comparable to ours, as the exuvial fluid of lepidopteran or coleopteran larvae and pupae originates from hypodermal/Verson’s glands that are inserted within the abdominal epidermis, while in other species, this fluid originates from rectal glands, they highlight the role of exuvial fluid in providing innate immunity and providing functions required during molting. Nonetheless, the proteomic analyses here support the view that, in addition to protecting the organism from infection, a synergistic function of the SG apocrine secretion is to enable exuvial fluid to facilitate molting.

**Figure 2:**
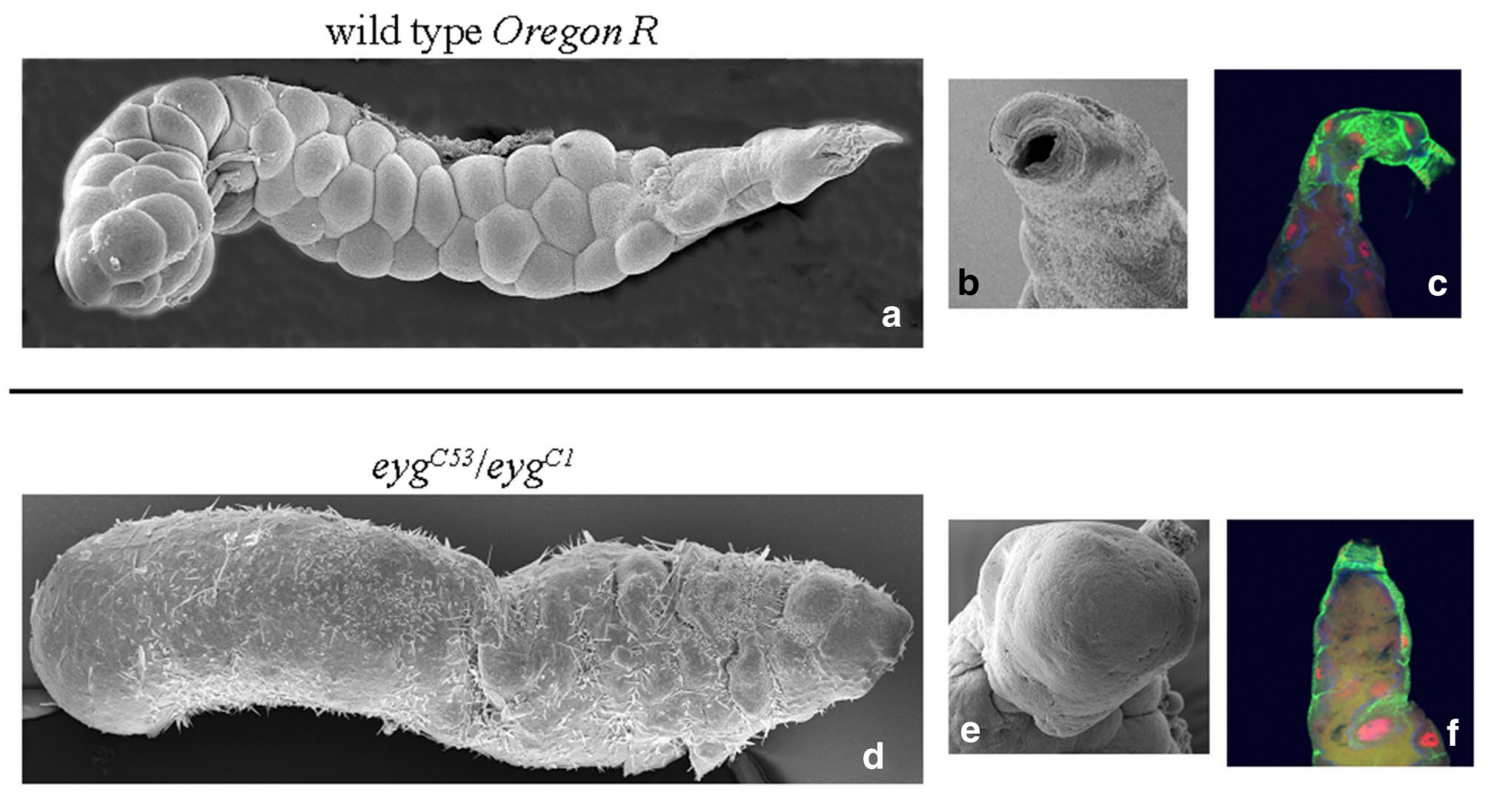
SG phenotype in *eyg*^*C1*^/*eyg*^*C53*^ mutants. (**a**) Longitudinal SEM view of the wild-type (*Oregon R*) SG at the end of the 3rd instar, with (**b**) detailed perpendicular view of its opening into the SG duct. (**c**) Laser confocal image of the anterior end of a wild-type 3rd larval instar SG showing a few columnar cells and the duct [tumor suppressor protein p127^l(2)gl^ (green), nuclear transcription factor BR–C (red), and filamentous actin (blue)]. (**d**) SEM view of a *eyg*^*C1*^/*eyg*^*C53*^ mutant salivary gland, with (**e**) a detailed perpendicular view of the mutilated, closed anterior end of the gland lacking a duct. (**f**) Optical section of the anterior end of *eyg*^*C1*^/*eyg*^*C53*^ mutant 3rd larval instar SG showing that it is a closed sack having a few columnar cells and is missing a duct (channels as above).

**Figure 3:**
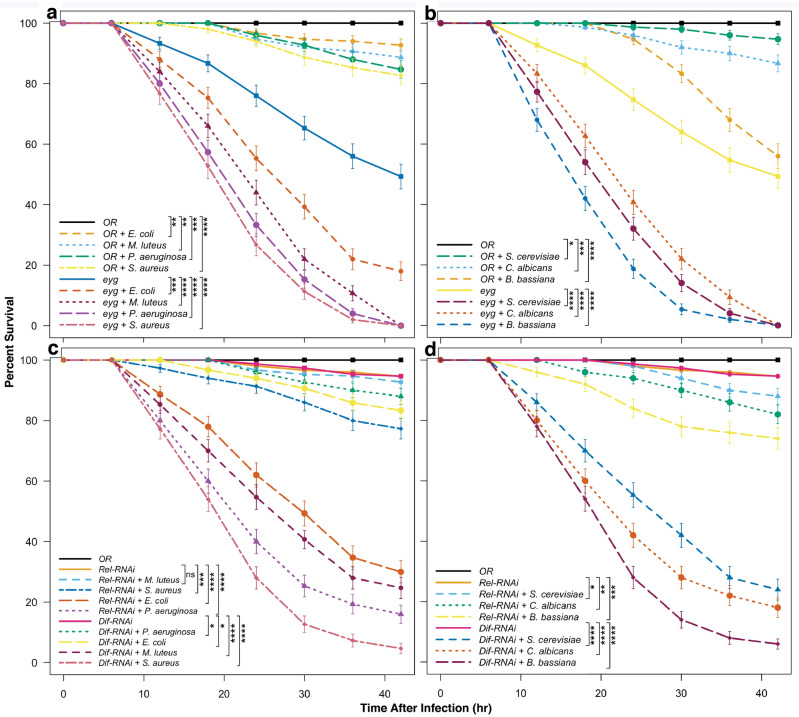
Pupae unable to deliver an apocrine secretion or with impaired Dif or Rel signaling during the late-larval and prepupal period are more susceptible to microbial infection. Mutant *eyg*^*C1*^/*eyg*^*C53*^ pupae, who lack a SG duct to deliver the apocrine secretion, show reduced survival when challenged with Gram-negative (*E*. *coli* and *P*. *aeruginosa*) or Gram-positive (*M*. *luteus* and *S*. *aureus*) bacteria (**a**) or when challenged with yeasts or fungi (*S*. *cerevisiae*, *C*. *albicans* and *B*. *bassiana*) (**b**). Pupae with impaired Dif (*Sgs4*-*Gal4* >  > *UAS*-*Dif*^*RNAi*^) or Rel (*Sgs4*-*Gal4* >  > *UAS*-*Dif*^*RNAi*^) signaling show reduced survival when challenged with Gram-negative (*E*. *coli* and *P*. *aeruginosa*) or Gram-positive (*M*. *luteus* and *S*. *aureus*) bacteria (**c**). Pupae with compromised Rel expression are more sensitive to Gram-negative than Gram-positive bacteria, while those with compromised Dif expression are more sensitive to Gram-positive than Gram-negative bacteria. (**d**) Pupae with impaired Dif or Rel signaling have diminished survival when challenged with yeasts or fungi (*S*. *cerevisiae*, *C*. *albicans* and *B*. *bassiana*). Data shown are the means ± SEM of three independent experiments. Differences in survival were evaluated using the Mantel-Cox log-rank test: ns = not significant, *p < 0.05, **p < 0.001, ***p < 1 × 10^–5^, ****p < 1 × 10^–10^.

In order to functionally verify the immune and defensive role of the apocrine secretion, we generated mutant *Drosophila* larvae where its delivery was blocked. Mutations in the Pax-gene *eye gone* (*eyg*) cause an absence of the individual ducts of embryonic and larval SGs^21^. Scanning electron microscopy (SEM) revealed that the SG duct in loss-of-function (*eyg*^*C1*^ deficiency homozygotes; hypomorphic *eyg*^*C1*^/*eyg*^*C53*^ compound heterozygotes) *eyg* animals is mutilated, so that their SG becomes a sack unable to release either the Sgs-glue or the apocrine secretion. Since the secretory products remain confined within the SG proper (Fig. 2), these animals cannot deliver the apocrine secretion to the periexuvial space and lack exuvial fluid. To directly test whether the apocrine secretion serves an immune-defense role, we evaluated the survival of *eyg*^*C1*^/*eyg*^*C53*^ early pupae (15–16 h APF) to microbial challenges. We used Gram-negative (*Escherichia coli* and *Pseudomonas aeruginosa*) and Gram-positive (*Staphylococcus aureus* and *Micrococcus luteus*) bacteria, yeasts (*Saccharomyces cerevisiae* and *Candida albicans*) and entomopathogenic fungi (*Beauveria bassiana*). *B*. *bassiana* is a naturally occurring entomopathogenic fungus with pathological consequences even in wild-type pupae. All of these microorganisms significantly increase the lethality of *eyg*^*C1*^/*eyg*^*C53*^ pupae compared to wild-type (*Oregon R*) controls or mock-treatments (Fig. 3a,b). Although *eyg*^*C1*^/*eyg*^*C53*^ mutants generally have decreased survival relative to untreated or even microbially challenged wild-type pupae, microbial challenges to these mutants lead to significant lethality at 35 h postinfection. Lethality at this time is distinct from the developmentally-linked lethality seen in uninfected *eyg*^*C1*^/*eyg*^*C53*^ mutants. Except for a small percentage of escapers that survive until adulthood, lethality in these mutants occurs later, at the end of the pupal stage when the exuvial fluid is necessary for the transition to the early-pharate adult stage^21^. Therefore, animals have strongly reduced survival when exposed to microbial infection if the apocrine secretion containing innate-immunity factors is absent from the periexuvial space during the early to mid-pupal period. This provides experimental support for the contention that, while the puparial case serves as a structural barrier, the SG apocrine secretion provides the primary defense against microbial invasion during the pupal period.

We were concerned that the developmentally-linked lethality in *eyg*^*C1*^/*eyg*^*C53*^ mutant pupae may have contributed to their increased lethality following microbial challenges. Therefore, we directly tested the role of immune components known to be chiefly involved in the antimicrobial response in *Drosophila*^22–24^ by using RNAi to compromise the *Toll* and *immune deficiency* (*imd*) pathways. Since previous^25^ and our own studies indicated that the production of antimicrobial peptides in tissues other than hemocytes and the fat body are constitutive and non-inducible, we did not manipulate the *Toll* and *imd* genes directly. Rather, we targeted *dorsal immune*-*related factor* (*Dif*) and *Relish* (*Rel*), which encode downstream components of the *Toll* and *imd* pathways. Their products act as transcription factors controlling expression of antibacterial and antifungal peptides in *Drosophila*^20,26,27^. Dif primarily controls transcription of antimicrobial peptides against Gram-positive bacteria and fungi, whereas Rel controls transcription of antimicrobial peptides specific against Gram-negative bacteria^26–29^. Therefore, we drove *UAS*-*Dif*^*RNAi*^ and *UAS*-*Rel*^*RNAi*^ constructs using *Sgs4-Gal4*, so that the production of antimicrobial peptides was compromised only in SGs. Microbial challenges decreased survival of both *Dif*- and *Rel*-compromised genotypes (Fig. 3c,d), albeit less than in *eyg*^*C1*^/*eyg*^*C53*^ pupae. Also, lethality in *Dif*- and *Rel*-knocked down pupae was delayed some 5 to 7 h in comparison to *eyg*^*C1*^/*eyg*^*C53*^ mutants. These differences are consistent with the expectation that *Dif*- and *Rel*-compromised genotypes only partially ablate the immune complement of the apocrine secretion. Indeed, pupae with knockdown of Rel function had increased susceptibility to Gram-negative bacteria while pupae with knockdown of Dif function had increased susceptibility to Gram-positive bacteria. The delay in the lethality of these animals relative to *eyg*^*C1*^/*eyg*^*C5*^ mutants also could arise from the presence of non-proteinaceous germicidal agents, such as oxalate salts, which would remain present in the apocrine secretion^4,30^. Altogether, these experiments show that pupae with SGs compromised of antibacterial and/or antifungal peptides lose their corresponding antimicrobial defenses and confirmed that the SG apocrine secretion is a crucial source of immune factors for exuvial fluid to defend against microbial invasion.

**Figure 4:**
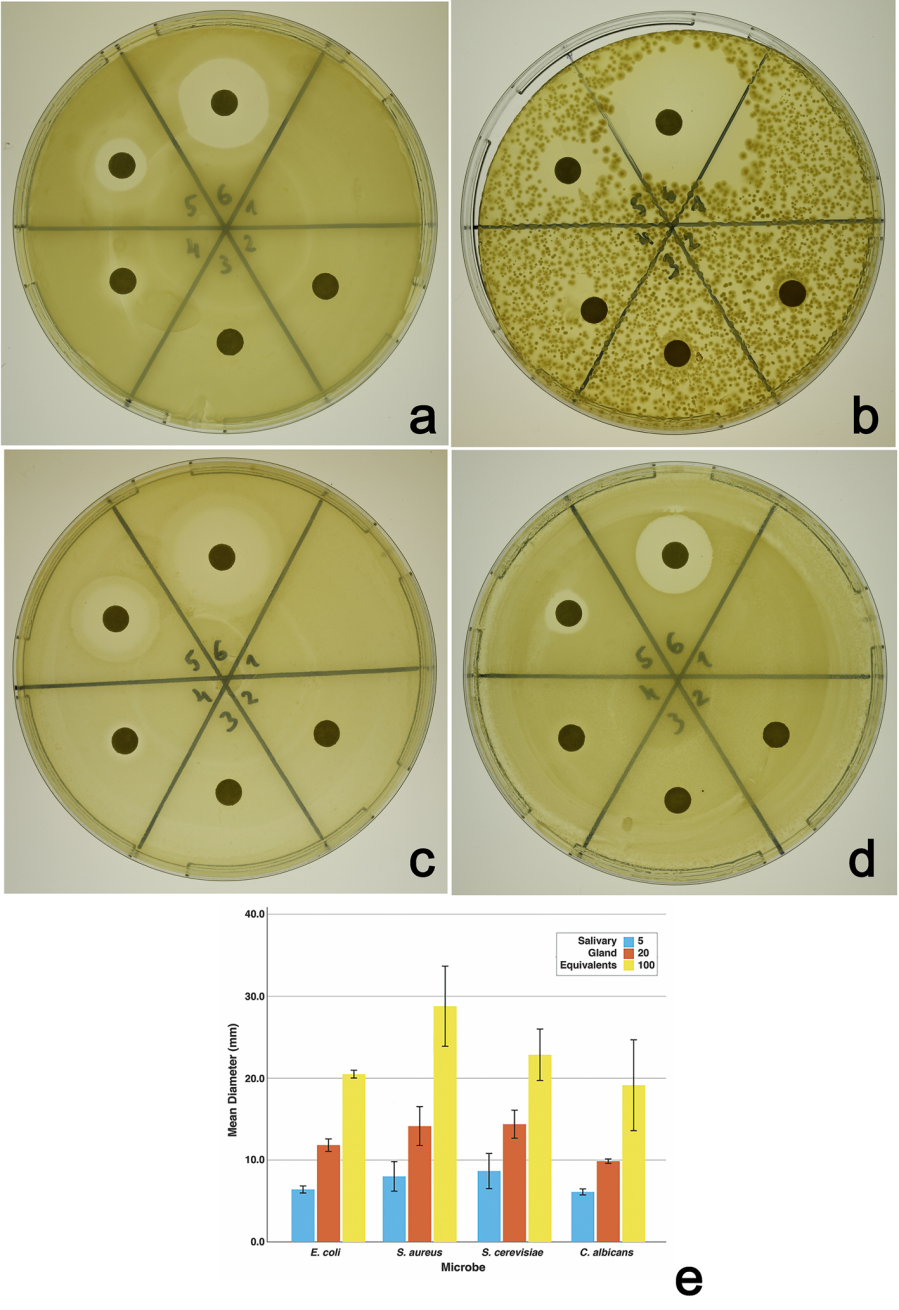
Germicidal properties of the proteinaceous apocrine secretion isolated from the lumen of late prepupal SGs. Apocrine secretion from the equivalent of 5, 20 or 100 wild-type SGs was applied in a 10 μl volume of sterile PBS onto 6-mm diameter Rotilabo filters placed on 10-cm LB-agar (**a,b**) or YPD-agar (**c,d**) plates previously spread with *E*. *coli* (**a**), *S*. *aureus* (**b**), *S*. *cerevisiae* (**c**), or *C*. *albicans* (**d**). Sectors 1, 2, and 3 had no filter, an untreated filter, or a filter with only PBS, respectively. Sectors 4, 5, and 6 had filters with the apocrine secretion from the equivalent of 5, 20, or 100 pairs of SGs, respectively. Greater amounts of the apocrine secretion were associated with increased growth inhibition for each microbe tested. Images show data from one representative experiment. The graph shows the mean diameter of growth inhibition, measured using Image J, observed in three replicate experiments (Error bars: 95% CI). There were statistically significant differences in the degree of growth inhibition of each microbe by different amounts of SG extract as determined by one-way ANOVA (*E*. *coli*:*F*(2,6) = 2788, *p* = 1.2e−09; *S*. *aureus*: *F*(2,6) = 192.8, *p* = 3.6e−06; *S*. *cerevisiae*: *F*(2,6) = 162.4, *p* = 6.0e−06; *C*. *albicans*: *F*(2,6) = 80.6, *p* = 4.6e−05). For each microbe, post-hoc TukeyHSD tests revealed significant differences (adjusted *p* < 0.05) in the mean growth inhibition by different amounts of SG extract in all pairwise comparisons. Differences in growth inhibition of Gram-negative (**a**) and Gram-positive (**b**) bacteria may partly reflect differences in culture density.

Finally, we directly tested whether the apocrine secretion possessed antimicrobial activity. We isolated the apocrine secretion from the lumen of prepupal SGs from wild-type (*Oregon R*) animals^9^, spotted the secretion-equivalent of 5, 20 or 100 SG-pairs in a 10 μl volume onto the surface of 6-mm Rotilabo circle filters, and placed the filters on LB- or YPD-agar media having freshly spread bacteria (*E*. *coli*, *S*. *aureus*) or yeast (*S*. *cerevisiae* and *C*. *albicans*). While no treatment and filters without any apocrine secretion do not inhibit microbial growth, the SG secretion inhibits microbial growth in a dose-dependent manner (Fig. 4). In these ex vivo tests, the apocrine secretion extracts are slightly more effective against Gram-positive than Gram-negative bacteria or yeasts but this difference, compared to the in vivo response of wild-type pupae (Fig. 3a,b), may reflect the more optimal growth of these microorganisms on defined media.

## Discussion

Individual proteins in the apocrine secretion/exuvial fluid often function in multiple processes (Supplemental Tables S2, S3). Indeed, an analysis using the Search Tool for the Retrieval of Interacting Genes (STRING) revealed proteins form a functional protein–protein interaction network. Proteins functioning in antimicrobial defense and molting also have functional interactions with each other and/or proteins involved in other homeostatic, metabolic, cellular and/or developmental processes (Supplemental Figures, Supplemental Tables S2–S4).

In *Drosophila,* the SGs are the major apocrine secretory organ, and the larval/prepupal SGs are the only source of pupal exuvial fluid. In this work, we show that abrogation of the SG apocrine secretion or knockdown of antimicrobial peptides within it decreases pupal survival to microbial challenges, and ex vivo testing demonstrates that the prepupal SG apocrine secretory fluid possesses antibacterial and antifungal activity. While the exuvial fluid does not contain any elements of non-specific cellular immunity which would support its defense response^31^, proteomic analysis reveal that it contains numerous proteins used in innate immunity, as well as proteins required for molting.

Molecular genetic approaches using the *Drosophila* SG system will be invaluable to gain insight into the mechanisms used to regulate apocrine secretion. Indeed, once sufficient insight into the molecular regulation of apocrine secretion is gathered, it will become possible to specifically block apocrine secretion in the *Drosophila* system. This will allow for confirmation of the main inference of this work: that apocrine secretion, and not some other as-yet undefined process, is responsible for exporting the components of innate immunity into the SG lumen for future delivery to the exuvial fluid.

If SG apocrine secretion plays a critical role in providing innate immunity, it should be conserved across different *Drosophila* species. We screened 30 species of *Drosophila* from various geographical regions and habitats of five continents and found that, unlike the exocytotic secretion of Sgs-glue, apocrine secretion is evolutionarily conserved (Farkaš et al. in preparation). Thus, the *Drosophila* SG is a typical labial gland that provides a set of species- and very possibly genus- or infraorder-specific functions. These results transform our view of the role of the SG in the metamorphosis of holometabolous insects: they show that SG apocrine secretion delivers fully immunocompetent exuvial fluid, contributing an antimicrobial moat lying between the defensive rampart deployed by the pupal case and the pupa itself.

Innate immunity seems to be a common function provided by apocrine secretion. Comparison of the SG-apocrine-secretion proteome to that of secretions from human apocrine glands (sweat, mammary, cerumenal, lacrimal, etc.)^9,12,13^ reveals not only that the major ontological categories of proteins are evolutionary highly conserved, but that all of these apocrine secretions share components of an innate immune response. Therefore, apocrine secretion serves as an evolutionarily conserved mechanism to deliver innate immune components ready for an immediate defense response.

## Materials and methods

### Fly culture, staging and genetics

Flies were cultured in 50 ml glass vials or 200 ml bottles at 23 °C on agar-yeast-cornmeal-molasses medium^32,33^ with the addition of methylparaben to prevent molds. Observations were carried out on last (3rd) instar larvae of *Drosophila melanogaster* (Meigen) and the wild type strain *Oregon R*, originally obtained from Umea Drosophila Stock Centre, Umea, Sweden, was used as standard reference control^34^.

Two mutations in the Pax gene *eye gone* (*eyg*) were used: *Df(3L)eyg*^*C1*^ (69A4-5; 69D4-6), which deletes *eyg*, and *In(3L)eyg*^*C53*^ (69B4-C7), which breaks in or just adjacent to the *eyg*-coding region^21,35^. These mutations were gift of Steve Beckendorf (University of California, Berkeley) and Y. Henry Sun (Academia Sinica, Taipei, Taiwan). Besides its profound effect on head and eye development, the *eyg* gene is also required for salivary gland (SG) duct formation. Its mutations cause ablation of both the common and the individual SG ducts. Although homozygous *eyg*^*C1*^ animals die as embryos, *eyg*^*C1*^/*eyg*^*C53*^ animals hatch, pupariate and pupate. However, only a few of them survive to adulthood. With respect to their SG duct phenotypes, *eyg*^*C53*^ behaves as a hypomorph, as the phenotype of *eyg*^*C1*^/*eyg*^*C53*^ embryos is more severe than that of *eyg*^*C53*^ homozygous embryos. The *TM6B*, *Tb* or *TM6C*, *GFP Tb* balancer chromosomes were used to distinguish *eyg* larvae and pupae from their wild-type siblings.

The *Sgs4*-*Gal4* is a driver line containing the tissue- and stage-specific promoter sequences of the *Sgs4* gene (from − 840 to + 1 bp upstream regulatory region) fused to the *Gal4* activator coding sequence^36,37^ and rendering stage- and tissue-specific high level expression of any *Gal4*-driven *UAS*-target sequence in the late third-instar larval and early prepupal SGs^38–40^. The *Sgs4*-*Gal4* transgenic construct was a gift of Annemarie Hofmann (Free University, Berlin, Germany).

The *Sgs3*-*GFP* construct is a fusion of the jellyfish GFP coding sequence behind the 1.8 kb of the *Sgs3* gene carrying its upstream regulatory information and the first third of its protein coding sequence truncated after nine tandem repeats^11^. These sequences were fused in-frame into *pCaSpeR-4* vector^41^ and used to generate transformed flies^42^. The *Sgs3*-*GFP* stock was obtained from Andy J. Andres (University of Nevada, Las Vegas, NV, USA).

The knock-down experiments using in vivo RNA interference (RNAi) for *dorsal immune*-*related factor* (*Dif*) and *Relish* (*Rel*) were performed with *UAS*-*Dif*^*RNAi*^ and *UAS*-*Rel*^*RNAi*^ constructs in the VALIUM 10 vector^43,44^ expressed under control of the *Sgs4-Gal4* driver described above. For *UAS*-*Dif*^*RNAi*^, the *y*^*1*^* v*^*1*^; *P*{*TRiP*.*HM05191*}*attP2* stock (B-29514) was used; this line has been shown to efficiently knock down expression of *Dif* in several previous studies^45–50^. For *UAS*-*Rel*^*RNAi*^, the *y*^*1*^* v*^*1*^; *P*{*TRiP*.*HM05154*} *attP2* stock (B-28943) was used; this line has been shown to efficiently knock down expression of *Rel* in several previous studies^45,49,51–54^. Confirmation of knock-down was evaluated as shown in Supplemental Fig. 3. These fly stocks were obtained from Bloomington Drosophila Stock Center, Indiana University, Bloomington, IN, USA.

Newly formed white puparia were considered to be 0 h PP; when necessary, SGs were dissected from PP hourly after puparium formation (APF) as described elsewhere^55^. The majority of the described experiments used SGs 8–10 h APF.

Whole animal imaging of GFP fluorescence was performed on Leica MZ16F A/X fluorescent stereomicroscope equipped with a DFC480 digital camera using Leica LAS 2.6.0-R1 software and the Multifocus function of the Montage module utilizing an orthogonal alignment method.

### Tissue dissection

For the experiments described herein, salivary glands were dissected from the prepupae using extrafine Dumont # 5 tweezers while they were viewed under a Wild M3Z or Leica MZ9,5 stereomicroscope in *Drosophila* saline solution. They were then gently rinsed three times in clean *Drosophila* saline, and processed either for microscopy or used to collect apocrine secretion form their lumen (see below).

### Immunohistochemistry and confocal microscopy

Dissected SGs were fixed in PIPES-buffered 4% paraformaldehyde (20 mM PIPES, 60 mM sucrose, 1 mM EGTA, 5 mM MgCl_2_, pH 7.2). In order to stain tissue with antibodies the SGs were permeabilized with 0.1% Triton X-100 in PBS (PT) and then blocked with PT containing 2% fraction V of bovine serum albumin (Serva) (PBT) and 2% goat serum (Sigma) (PBTS). To detect nuclear, cytosolic and cytoskeletal proteins, the SGs were incubated overnight at 4 °C in PBTS in the presence of primary mouse anti-BR-C monoclonal antibody^55–57^ diluted 1:10 and primary rabbit anti-p127^l(2)gl^ polyclonal antibody^55,58^ diluted 1:200. After extensive washing of the SGs in PT, goat affinity purified and preadsorbed Cy5-anti-mouse and Cy3-anti-rabbit F(ab)_2_-specific secondary antibodies (Jackson ImmunoResearch Labs.) were added along with 0.04 nM AlexaFluor_488_-Phalloidin (Molecular Probes/Invitrogen) and 5 μg/ml Hoechst-33258 (Calbiochem), and the SGs were incubated for 2 h at room temperature (23 to 25 °C). After extensive washing in PT, the salivary glands were mounted in Elvanol under a Schött high-performance No 1.5H coverslip and scanned under a Zeiss LSM-510 META laser confocal microscope using a 40 × (NA 1.3) oil objective lens. The obtained bitmap images were processed using Zeiss AIM LSM5 software and Adobe Photoshop.

### Scanning electron microscopy (SEM)

Whole salivary glands were fixed immediately after dissection in 4% paraformaldehyde (Polysciences) + 2% glutaraldehyde (Ted Pella) in 0.1 M sodium cacodylate (Serva) (pH 7.2) for 1 h at 23 to 25 °C. To ensure they were fully immersed in fixative, the SGs were placed in inverted caps from 1.5 ml eppendorf tubes and ~ 100 μl of fixative was used per 10 pairs of glands. The SGs were then rinsed 5× in 100–200 μl of fresh 0.1 M sodium cacodylate (pH 7.2) for 5 to 10 min each at 23 to 25 °C. The samples were postfixed in 0.5% osmium tetroxide (EMS) in H_2_O for 60 min, and then extensively rinsed in H_2_O (minimum 6× for 10 min each). The SGs were dehydrated in an ascending series of ethanol (Merck) (30%, 50%, 70%, 96% and 100%); the dehydration step with 100% ethanol was repeated twice before applying a mixture of 100% ethanol + 100% acetone (Merck) (1:1) twice, followed by three changes of absolute acetone. At this step care was taken to prevent the sample from drying completely during the exchanges of absolute ethanol or acetone, and at all subsequent times before critical point drying. Therefore, the addition of hexamethyldisilazane (HMDS; Sigma-Aldrich), in place of Peldri II^59–61^ to facilitate critical point drying^62,63^, was done carefully in several steps: the first volume of HMDS was applied in the presence of small remnants of acetone; then, after 5 to 10 min, the HMDS was quickly removed and fresh HMDS added taking care that previous HMDS did not completely evaporate; finally, SGs were then kept in the HMDS for 30 min, and then the remnants of HDMS were allowed to evaporate completely in a dust-free space.

Dried SGs mounted on pieces of Scotch double-sided tape on 16 or 24 mm aluminum SEM stubs were sputter coated for 2.5 min with gold–palladium using a Balzers sputter-coater device SCD-030 at 35–40 mA per stub and at a pressure of 0.05 to 0.1 mbar to produce a 40–50 nm continuous alloy layer. The samples were viewed and photographed on an FEI Quanta FEG250 scanning electron microscope with the emission field cathode at an accelerating voltage of 10 kV. The bitmap images obtained were processed and labeled using Adobe Photoshop or Corel Draw software and assembled into figures using Adobe Photoshop.

### Isolation and collection of apocrine secretion and exuvial fluid

Twenty pairs of prepupal salivary glands from animals 8–10 h APF were quickly dissected in sterile *Drosophila* saline (as described above) during a single session. A SG was individually transferred to a fresh 10 μl drop of sterile PBS. The salivary gland was then carefully and gently squeezed along its longitudinal axis, starting at most posterior end, with a No. 5 Dumont extrafine or Moria superfine tweezers to apply delicate pressure able to expel the luminal contents into the PBS drop without disrupting the cells of the SG. To ensure that the SG cells were not disrupted, this process was monitored using a good stereomicroscope (Leica MZ9.5 or MZ12) with adjustable bright field transillumination (Wild M5A or M420 “Durchlichtstative” base). The squeezed gland was immediately removed from the drop, a new gland was added to the same drop, and the process was repeated until secretions from twenty glands were obtained. As specified below, the collected secretions were used either for testing antimicrobial activity or for protein extraction and analysis.

To obtain exuvial fluid, twenty pupae of the appropriate age (5 to 7 h old pupae [i.e. 18 to 20-h APF]) were collected, their puparial cases were cleaned for 3 min with tap water, then briefly cleaned with diluted dishwashing liquid (e.g. Dawn Ultra or Palma Jar) to remove any surface contaminants, and again rinsed for 3 min with tap water, and then rinsed with d H_2_O by exchanging the water five times, decanting the water each time, and then air-dried. Cleaned pupae were transferred into 50 μl of sterile PBS containing the protease inhibitor cocktail (1 mM bestatin, 100 μM chymostatin, 7.5 μM antipain, 1 μM leupeptin, 50 μg/ml AEBSF, 1 mM phenylmethylsulfonylfluorid, 1 μM aprotinin, 10 μM benzamidine, 8 μM phosphoramidone and 20 μg/ml E64; components from Calbiochem, Roche and Sigma), and individual pupae carefully opened with a No. 5 Dumont extrafine tweezers without injuring the enclosed animal. Opened animals were allowed to stay in the drop of PBS for 10 to 15 min to facilitate the free diffusion of the exuvial fluid into the drop. The collected exuvial fluids were then frozen in dry ice and stored at − 80 °C until being pooled and used for downstream analysis.

### Microbial infection assays

The following bacterial and yeast strains were used: *Escherichia coli* (K12), *Pseudomonas aeruginosa* (PA14), *Staphylococcus aureus* (RN6390), *Micrococcus luteus* (ATCC4698), *Saccharomyces cerevisiae* (BY4741) and *Candida albicans* (SC5314), and entomopathogenic fungi *Beauveria bassiana* (80.2). *E*. *coli* were grown in Luria–Bertani broth^64^* P*. *aeruginosa* and *S*. *aureus* were grown in Bushnell-Haas broth, *S*. *cerevisiae* were grown in YPD media^64^, *C*. *albicans* were grown in Sabouraud broth, and *B*. *bassiana* were grown in TKI broth. Bushnell-Haas broth and Sabouraud broth were prepared according to Difco manual. TKI broth was prepared according to Thomas et al.^65^ with modifications of Lohse et al.^66^. Except salts, dextrose, fructose or maltose (Sigma or Fisher Scientific), all other components of these media were of Bacto-grade purchased from Difco/Becton–Dickinson Corp.

Bacterial and yeast/fungal infections in pupae were generated by pricking the puparial case of 2–3 h old pupae (15–16 h APF). Great care was taken to prick only the puparial case at the most anterior empty space, the space left after head eversion occurs. This was done while viewing the animal under a stereomicroscope and by using an extra sharp tungsten needle previously dipped into a concentrated pellet of a microbial culture (OD_200_). The size of the test group for each genotype, including control wild type (*Oregon R*) animals, consisted of 50 pupae. After pricking, the animals were gently shaken for 30 s in a filter paper-lined 5 cm plastic Petri dish to facilitate a more or less regular distribution of the inoculum. Survival experiments were carried at 25 °C, and pupal survival was scored by counted survivors every 6 h for the following 50 h. The data sets shown in Fig. 3 are representative of at least three independent experimental replicates. Results are expressed as percentage of infected animals (survival rate) at different time points after infection.

### Ex-vivo testing of antimicrobial effects of the apocrine secretion

To test the antimicrobial potential of apocrine secretion ex vivo, it was isolated from dissected SGs into sterile PBS as described above. Then, a mixture of the apocrine secretion and PBS was applied in a 10 μl volume onto the surface of 6 mm Rotilabo circle filters (C. Roth GmbH., Germany) and these were placed on LB- or YPD agar in 9 cm polystyrene Petri plates that were freshly spread with a 200 μl inoculum of an overnight microbial culture. The LB and YPD media were prepared as above with the addition of Bacto-agar to 1.5% concentration. Empirically, we found that even though liquid culture is optimal for *S*. *aureus* using Bushnell-Haas broth and for *C*. *albicans* using the Sabouraud broth, these microorganisms grew well on solid agar-based LB and YPD media, respectively. Agar plates were incubated overnight at 37 °C (for bacteria) or 36 h at 29 °C (for yeasts), and subsequently examined and photographed to document the inhibition of microbial growth. Each data set (agar plate) shown in Fig. 4 is representative of at least three independent experiments.

### Proteomics analysis

#### Sample collection and electrophoresis

For proteomic analysis, 20 pairs of prepupal SGs (a single-session’s collection) from animals 8–10 h APF were dissected as described above and transferred to a fresh 10 μl drop of sterile *Drosophila* saline (instead of PBS) containing the protease-inhibitor cocktail (1 mM bestatin, 100 μM chymostatin, 7.5 μM antipain, 1 μM leupeptin, 50 μg/ml AEBSF, 1 mM phenylmethylsulfonylfluorid, 1 μM aprotinin, 10 μM benzamidine, 8 μM phosphoramidone and 20 μg/ml E64; components from Calbiochem, Roche and Sigma). Each SG was carefully and gently squeezed along its longitudinal axis as described above to expel the luminal contents into the *Drosophila* saline drop without disrupting SG cells. This process was closely monitored using a stereomicroscope with adjustable bright field transillumination (see above). After the luminal contents of all 20 pairs of glands were pressed out, the drop of *Drosophila* saline with the secreted material was immediately transferred to a clean Lo-Bind Eppendorf tube and 10 μl of SDS-sample extraction buffer (12.5 mM Tris–HCl, 2% SDS, 5% β-mercaptoethanol, 10% glycerol pH 6.8 plus protease inhibitors cocktail) added. The sample was extracted for 5 min at 100 °C, centrifuged at 20,000×*g* for 15 min at room temperature and the supernatant frozen at − 80 °C. During these and all subsequent steps, extreme care was taken to avoid any air-born contamination of the samples (dust, bacteria, human skin etc.). Upon thawing, protein extracts from 200 gland pairs (10 independent extractions of 20 pairs each) were quickly pooled and loaded onto a 10% polyacrylamide-SDS (SDS-PAGE) gel and electrophoresed at a constant current of 20 mA for ~ 3 h or until the front of the Schlieren line reached the bottom of the gel^9^. The gel with the separated proteins was fixed in 50% methanol and 10% acetic acid for 1 h and stained with Coomassie brilliant blue R-250 (Serva), or PageBlue protein dye (Fermentas). The gel was allowed to destain in 5% methanol and 7% acetic acid for a minimum of 4 days (with several exchanges of the solute). If not used for SDS-PAGE, thawed samples were precipitated with 3 volumes of cold methanol, followed by a chloroform clean-up step, and centrifuged at 20,000 × *g* for 10 min. After the liquid was carefully removed, the pellet was dried under vacuum (Speed Vac)^67^.

### Enzymatic in-gel digestion and chemical derivatization

Unless otherwise stated, for all downstream and below described applications used Milli-Q (18 MΩ or 0.05 μS/cm) ultra pure water, and most of the chemicals used were of proteomic or mass-spec grade from Merck-Sigma-Aldrich, Fluka or J.T. Baker. Electrophoretically separated protein bands were excised from the gel with a sterile ophthalmological scalpel and transferred to a Protein Lo-Bind microtube (Eppendorf) and rinsed three times with sterile deionized water. The Coomassie stain was removed by repeated washing in 50 mM NH_4_HCO_3_ and 50% acetonitrile. The proteins in the gel pieces were first reduced with 10 mM dithiothreitol (DTT), and then alkylated with 55 mM iodoacetamide. In-gel digestion was performed using 1 mM sequencing-grade gold trypsin (Promega) at 37 °C for 12 h and subsequently acidified with 5% formic acid. Peptide extraction from the digest was done according to Shevchenko et al.^68^ and the extracts were dried down using an Eppendorf 5301 centrifugal vacuum concentrator at 30 °C. The recovered peptides were dissolved in 50 μl of 0.1% TFA. Further purification was achieved by C_18_ ZipTip pipette tips (Millipore, Bedford, MA, USA) used according to the manufacturer's instructions. Samples prepared this way were used for MALDI-TOF/TOF as well as ESI–MS/MS analysis.

### MALDI-TOF/TOF mass spectrometry

MALDI-TOF/TOF mass spectrometry was performed as described previously^9^. Briefly, a saturated solution of α-cyano-4-hydroxycinnamic acid (CHCA) (Sigma) in 50% acetonitrile/0.1% TFA (Fluka AG) was used as a MALDI matrix. The protein mixture was spotted onto the MALDI target plate and allowed to dry at 25 °C. MALDI-TOF/TOF and MS/MS mass spectra were obtained in the positive ionization mode using ABI 4700 and ABI 4800 Proteomics Analyzers (Applied Biosystems) equipped with a solid state laser (diode pumped Nd:YAG laser) pulsing at a repetition rate of 200 Hz (pulse duration < 500 ps) and operating at a wavelength of 355 nm. The spectra were acquired using a dual-stage reflectron mirror and accumulated from up to 2500 and 20,000 shots in MS and MS/MS mode, respectively. The instrument was calibrated externally using a mixture of five peptide standards. Accelerating voltages applied for MS and MS/MS measurements were 20 and 8 kV, respectively. In MS/MS mode, a collision energy of 1 kV was applied, and nitrogen was used as a collision gas in collision-induced dissociation experiments. Raw spectral data were further processed using DataExplorer 4.5 software (Applied Biosystems). Database searches were performed against non-redundant protein sequence databases (Uni-Prot, Trembl, MSDB and NCBI) using the program Mascot (Matrix Science Ltd.).

### Nano-liquid chromatography-tandem mass spectrometry analysis (nLC-ESI–MS/MS)

Two micrograms of protein tryptic digest were subjected to nLC-ESI–MS/MS analysis as published previously^69^. Briefly, spectral data were collected using an Orbitrap LTQ Velos mass spectrometer (Thermo Fisher Scientific, Waltham, MA, USA) linked with an UltiMate 3000 nano flow HPLC system (Thermo Fisher Scientific). Peptides were separated on a reversed phase Acclaim PepMap C18 column (Thermo Fisher Scientific) via a 170-min long nonlinear gradient of acetonitrile (in 0.1% formic acid) 2–55% for 125 min, 95% for 20 min with a constant flow rate of 300 nl/min. Ions were detected in the linear trap mass detector operated in a data dependent acquisition (DDA) mode with dynamic exclusion being applied, in 18 scan events: one MS scan (m/z range: 300–2000) followed by 17 MS/MS scans for the 17 most intense ions detected in the MS scan.

### Protein identification

The raw data files were searched using the SEQUEST algorithm of the Proteome Discoverer software version 1.1 (Thermo Fisher Scientific), as described previously^70^. Variable modifications were considered for: cysteine carbamidomethylation (+ 57.021), methionine oxidation (+ 15.995), and methionine dioxidation (+ 31.990). The target *Drosophila* protein database was acquired from the NCBI (www.ncbi.nlm.nih.gov). The reversed copy (created automatically by the software) served as a decoy database. To obtain high confidence protein identifications, the search results were filtered by FDR < 1%.

### Bioinformatic prediction of protein interactions

A protein–protein interaction network was predicted using the STRING^71,72^ database (version 11.0) applying a minimum required interaction score of 0.9 (high confidence prediction).

## Supplementary Information

Supplementary Information 1.

Supplementary Information 2.

## Data Availability

The data described in this manuscript is available within the manuscript and its Supplementary Information. Raw data on the proteomic analysis reported in the Supplemental Information is available by upon request from RF and does not require an MTA for access.
