# Supplementary material for: An apocrine mechanism delivers a fully immunocompetent exocrine secretion
Source: Sci Rep. 2021 Aug 5;11:15915. doi: 10.1038/s41598-021-95309-8 (PMC8342421; doi:10.1038/s41598-021-95309-8)
Supplement: Supplementary file 2 — Supplementary Information 2. [file 41598_2021_95309_MOESM2_ESM.doc]

Supplementary Information

**An Apocrine Mechanism Delivers a Fully Immunocompetent Exocrine Secretion**

Denisa Beňová-Liszeková^1^, Lucia Mentelová^1,2^, Klaudia Babišová^1^, Milan Beňo^1^, Tibor Pechan^3^, Bruce A. Chase^4^ and Robert Farkaš^1*^

^1^Laboratory of Developmental Genetics, Institute of Experimental Endocrinology, Biomedical Research Center, Slovak Academy of Sciences, Dúbravská cesta 9, 84505 Bratislava, Slovakia, ^2^Department of Genetics, Comenius University, Mlynská dolina, B-1, 84215 Bratislava, Slovakia, ^3^Institute for Genomics, Biocomputing and Biotechnology, Mississippi State University, 2 Research Boulevard, Starkville, Mississippi 39762, USA and ^4^Department of Biology, University of Nebraska at Omaha, 6001 Dodge Street, Omaha, NE 68182-0040, USA

Short title: Apocrine secretion provides systemic immunity

Author for correspondence: Robert Farkaš

Laboratory of Developmental Genetics

Institute of Experimental Endocrinology

Biomedical Research Center

Slovak Academy of Sciences

Dúbravská cesta 9

845 05 Bratislava

Slovakia

phone: (+421 2) 3229-5235

fax: (+421 2) 5477-4284

E-mail: ueenfark@savba.sk

**SUPPLEMENTAL TABLES**

**Supplemental Table 1**. List of proteins detected by mass spectrometry in the apocrine secretion (obtained from the lumen of prepupal salivary glands) and in the exuvial fluid (obtained from periexuvial space of early pupae, 18 - 20 hr APF).

All proteins are found in both the apocrine secretion and the exuvial fluid, though the scores, peptides identified, and coverage of some proteins differ (columns F/G, H/I, and J/K, respectively; SG = salivary glands and EF = exuvial fluid). These slight differences in the samples likely reflects the method of their collection. Many proteins are known, while others serve as basic components of exuvial fluid (column M). The antimicrobial factors are represented by drosomycin, cecropin A1, PGRP-SB2, poor Imd response upon knock-in, immune response deficient 1, ten Eig71E proteins, Immune-regulated catalase, Drosomycin-like 6, Neuropeptide-like precursor 2, NUCB1, peroxiredoxin 5, CG13551, apoptosis inducing factor, sickie, unpaired 1, and nimrod B2. Among them PGRP-SB2 is chiefly involved in surface recognition of infectious agens, while drosomycin, cecropin A1, Drosomycin-like 6, Eig71Es and others are directly acting antibacterial and/or antifungal*^25^*, *^28^*, *^73^*^-^*^77^*. On the other hand, poor Imd response upon knock-in, immune response deficient 1, Niemann-Pick type C-2a, unpaired 1 and sickie are examples of upstream signaling molecules or regulatory factors, and indirect effectors that lie within the innate immune response*^22^*, *^23^*, *^78^*^-^*^87^*. In addition, these proteins that serve canonical roles in the antimicrobial response, TER94 and MstProx represent factors known to be involved in an antiviral response*^23^*, *^88^*^-^*^92^*, thereby indicating that the apocrine secretion provides an even higher level of robustness for defensive reactions. It is possible that endonucleases (*e*.*g*. CG14062) present in the apocrine secretion are part of the non-specific antiviral artillery. An additional indicator of the complexity of the apocrine secretion is provided by various stress-response effectors and detoxification factors such as Atox1, superoxide dismutase, glutathione S transferase, CG12384, or CG3714*^93-96^*. Ferritin, especially ferritin 2 light chain, was already recovered in previous screens for systemic immune response in *Drosophila^94^*, *^97^*^-^*^99^*.

In addition, the apocrine secretion and exuvial fluid also includes several chitinases (Imaginal disc growth factor 3, Imaginal disc growth factor 4, CG8460), dipeptidyl aminopeptidase III and chitin-binding proteins (ghost, dumpy, CG5897) that all are typical representatives of exuvial fluid components. Furthermore, several peptidases and proteinases (*e*.*g*. matrix metalloproteinase 1, cysteine proteinase-1, ubiquitin-specific protease 7, proteasome 28kD subunit 1, CG15111, Neprilysin-like 8, proteasome beta2 subunit-related 2) as well peptidase/proteinase inhibitors (serpin, thiolester containing protein II, CG5384, crammer, Kazal serine peptidase inhibitors, cystatin-like, Kunitz proteinase inhibitor I2, CG42713, CG1440) could easily fulfill the function of exuvia-digesting enzymes or microbe-attacking hydrolases, if not both. Notably, peptidase/proteinase inhibitors are expected to play a role of impulse or signal switches that keep control over proteases and peptidases to avoid unwanted or precocious activation of a protease cascade unless their activity is needed, *e*.*g*. upon microbial challenge. Several other glycohydrolases and glycosyltransferases found in the apocrine secretion/exuvial fluid may be involved in digestion and processing of the chitin-based extracellular matrix that is required for moulting, an essential function of the exuvial fluid. The presence of mucin related 11Da and collagen type IV is expected to be chiefly associated with a role in lubrication*^100-102^* by the exuvial fluid that allows both delivery of the fluid and ensures apolysis.

It is striking that none of the immune-response related proteins is known to be inducible in the L3 or PP SGs upon infection*^23^*, *^25^*, *^73^*, *^103^*, though their induction is well documented inside the fat body, intestine, and hemolymph among other tissues*^22^*, *^26^*, *^27^*, *^99^*, *^104^*, *^105^*. However, based on FlyAtlas and modENCODE data*^106^*^-^*^109^* we found that several of these proteins are highly expressed in the late 3rd instar larval or prepupal SGs during normal development. This indicates that the SG does not respond immediately to a microbial challenge by triggering the production of antimicrobial peptides, but serves as a tissue for the specific storage of microbial peptides, and collects them in a reservoir to supply the future exuvial fluid.

Of the 125 proteins, 30 (24%) are proteins encoded by previously uncharacterized or poorly characterized *CG* genes, the function of which (except CG33998 and CG42713) could be estimated only via amino acid sequence similarity. In the several cases where no sequence similarity was identified, this work provides experimental evidence suggesting that they may function in antimicrobial defence.

**Supplemental Table 2**. STRING annotation of proteins identified in the apocrine secretion/exuvial fluid.

The 125 proteins described in Supplemental Table 1 were entered into STRING to evaluate their interactions in a functional network as described in the legend to the Supplemental Figure. STRING does not (always) distinguish between protein isoforms, so that 115 nodes were evaluated. Listed are the accession number used in the STRING analysis, the protein as named by STRING, the annotation provided by STRING, and the node color shown in the Supplemental Figure that identifies the broadly conceptualized process associated with the protein.

**Supplemental Table 3**. Processes associated with proteins of the apocrine secretion/exuvial fluid.

The table lists the STRING-generated gene ontology (GO) terms and process description, and the false discovery rate, IDs and names of proteins associated with each process.

**Supplemental Table 4**. PubMed IDs of literature supporting the functional assignments of proteins identified in the apocrine secretion / exuvial fluid.

Listed are the STRING-generated PubMed IDs of literature supporting the functional assignments of proteins within the functional protein-protein network, with their false-discovery rates. Additional citations supporting the functional assignments are presented in the information accompanying Supplemental Table 1.

**REFERENCES**

73. Ferrandon, D. *et* *al*. A drosomycin-GFP reporter transgene reveals a local immune response in *Drosophila* that is not dependent on the Toll pathway. *EMBO* *J*. **17**, 1217-1227 (1998).

74. Lindsay, S. A. & Wasserman, S. A. Conventional and non-conventional *Drosophila* Toll signaling. *Dev*. *Comp*. *Immunol*. **42**, 16-24 (2014).

75. Issa, N. *et* *al*. The circulating protease persephone is an immune sensor for microbial proteolytic activities upstream of the *Drosophila* Toll pathway. *Molec*. *Cell* **69**, 539-550 (2018).

76. Hanson, M. A. & Lemaitre, B. New insights on *Drosophila* antimicrobial peptide function in host defense and beyond. *Curr*. *Opin*. *Immunol*. **62**, 22-30 (2020).

77. Lu, Y. *et* *al*. Pattern recognition receptors in *Drosophila* immune responses. *Dev*. *Comp*. *Immunol*. **102**, 103468 (2020).

78. Fehlbaum, P. *et* *al*. Insect immunity: Septic injury of drosophila induces the synthesis of a potent antifungal peptide with sequence homology to plant antifungal peptides. *J*. *Biol*. *Chem*. **269**, 33159-33163 (1994).

79. Tauszig, S., Jouanguy, E., Hoffmann, J. A. & Imler, J. L. Toll-related receptors and the control of antimicrobial peptide expression in *Drosophila*. *Proc*. *Natl*. *Acad*. *Sci*. *USA* **97**,10520-10525 (2000).

80. Ooi, J. Y., Yagi, Y., Hu, X. & Ip, Y. T. The Drosophila Toll-9 activates a constitutive antimicrobial defense. *EMBO* *Rep*. **3**, 82-87 (2002).

81. Foley, E. & O'Farrell, P. H. Functional dissection of an innate immune response by a genome-wide RNAi screen. *PLoS* *Biol*. **2**, e203 (2004).

82. Kadrmas, J. L. *et* *al*. The integrin effector PINCH regulates JNK activity and epithelial migration in concert with Ras suppressor 1. *J*. *Cell* *Biol*. **167**, 1019-1024 (2004).

83. Kurucz, E. *et* *al*. Nimrod, a putative phagocytosis receptor with EGF repeats in *Drosophila* plasmatocytes. *Curr*. *Biol*. **17**, 649-654 (2007).

84. El Chamy, L., Leclerc, V., Caldelari, I. & Reichhart, J. M. Sensing of 'danger signals' and pathogen-associated molecular patterns defines binary signaling pathways 'upstream' of Toll. *Nat*. *Immunol*. **9**, 1165-1170 (2008).

85. Buchon, N. *et* *al*. A single modular serine protease integrates signals from pattern-recognition receptors upstream of the *Drosophila* Toll pathway. *Proc*. *Natl*. *Acad*. *Sci*. *USA* **106**, 12442-12447 (2009).

86. Cronin, S. J. *et* *al*. Genome-wide RNAi screen identifies genes involved in intestinal pathogenic bacterial infection. *Science* **325**, 340-343 (2009).

87. Valanne, S., Wang, J. H. & Rämet, M. The *Drosophila* Toll signaling pathway. *J*. *Immunol*. **186**, 649-656 (2011).

88. Nakamoto, M. *et* *al*. Virus recognition by toll-7 activates antiviral autophagy in *Drosophila*. *Immunity* **36**, 658-667 (2012).

89. Panda, D. *et* *al*. Genome-wide RNAi Screen Identifies SEC61A and VCP as Conserved Regulators of Sindbis Virus Entry. *Cell* *Rep*. **5**, 1737-1748 (2013).

90. Jiang, L. *et* *al*. Distinct functions of *Bombyx* *mori* peptidoglycan recognition protein 2 in immune responses to bacteria and viruses. *Front*. *Immunol*. **10**, 776 (2019).

91. Palmer, W. H. *et* *al*. Induction and suppression of NF-kappaB signalling by a DNA virus of *Drosophila*. *J*. *Virol*. **93**, e01443-18 (2019).

92. Sheehan, G., Farrell, G. & Kavanagh, K. Immune priming: the secret weapon of the insect world. *Virulence* **11**, 238-246 (2020).

93. Harrison, D. A., Binari, R., Nahreini, T. S., Gilman, M. & Perrimon, N. Activation of a *Drosophila* janus kinase (JAK) causes hematopoietic neoplasia and developmental defects. *EMBO* *J*. **14**, 2857-2865 (1995).

94. Levy, F., Bulet, P. & Ehret-Sabatier, L. Proteomic analysis of the systemic immune response of *Drosophila*. *Mol*. *Cell*. *Proteomics* **3**, 156-166 (2004).

95. Ortiz, J. G., Opoka, R., Kane, D. & Cartwright, I. L. Investigating arsenic susceptibility from a genetic perspective in *Drosophila* reveals a key role for glutathione synthetase. *Toxicol*. *Sci*. **107**, 416-426 (2009).

96. Radyuk, S. N., Michalak, K., Klichko, V. I., Benes, J. & Orr, W. C. Peroxiredoxin 5 modulates immune response in *Drosophila*. *Biochim*. *Biophys*. *Acta* **1800**, 1153-1163 (2010).

97. Kutty, R. K. *et* *al*. Molecular characterization and developmental expression of a retinoid- and fatty acid binding glycoprotein from *Drosophila*. A putative lipophorin. *J*. *Biol*. *Chem*. **271**, 20641-20649 (1996).

98. de Morais Guedes, S. *et* *al*. *Drosophila* *melanogaster* larval hemolymph protein mapping. *Biochem*. *Biophys*. *Res*. *Commun*. **312**, 545-554 (2003).

99. Vierstraete, E. *et* *al*. A proteomic approach for the analysis of instantly released wound and immune proteins in *Drosophila* *melanogaster* hemolymph. *Proc*. *Natl*. *Acad*. *Sci*. *USA* **101**, 470-475 (2004).

100. Fraichard, S., Bouge, A. L., Chauvel, I. & Bouhin, H. Tenectin, a novel extracellular matrix protein expressed during *Drosophila* *melanogaster* embryonic development. *Gene* *Expr*. *Patterns* **6**, 772-776 (2006).

101. Syed, Z. A., Härd T., Uv A. & van Dijk-Härd, I. F. A potential role for *Drosophila* mucins in development and physiology. *PLoS* *ONE* **3**, e3041 (2008).

102. Reis, M. A. Silva, C., Vieira, C. P. & Vieira, J. The *Drosophila* *melanogaster* Muc68E Mucin Gene Influences Adult Size, Starvation Tolerance, and Cold Recovery. *G3* (Bethesda) **6**, 1841-1851 (2016).

103. Capilla, A. *et* *al*. Toll pathway is required for wound-induced expression of barrier repair genes in the *Drosophila* epidermis. *Proc*. *Natl*. *Acad*. *Sci*. *USA* **114**, E2682-E2688 (2017).

104. Verleyen, P. *et* *al*. Identification of new immune induced molecules in the haemolymph of Drosophila melanogaster by 2D-nanoLC MS/MS. *J*. *Insect* *Physiol*. **52**, 379-388 (2006).

105. Carvalho, L., Jacinto, A. & Matova, N. The Toll/NF-κB signaling pathway is required for epidermal wound repair in *Drosophila*. *Proc*. *Natl*. *Acad*. *Sci*. *USA* **111**, E5373-E5382 (2014).

106. Chintapalli, V. R., Wang, J. & Dow, J. A. T. Using FlyAtlas to identify better *Drosophila* *melanogaster* models of human disease. *Nature* *Genet*. **39**, 715-720 (2007).

107. Graveley, B. R. *et* *al*. The developmental transcriptome of *Drosophila* *melanogaster*. *Nature* **471**, 473-479 (2011).

108. Robinson, S. W., Herzyk, P., Dow, J. A. T. & Leader, D. P. FlyAtlas: database of gene expression in the tissues of *Drosophila* *melanogaster*. *Nucleic* *Acids* *Res*. **41**, D744-D750 (2013).

109. Chen, Z. X. *et* *al*. Comparative validation of the *D*. *melanogaster* modENCODE transcriptome annotation. *Genome* *Res*. **24**, 1209-1223 (2014).

110. Žurovec, M., Doležal, T., Gazi, M., Pavlova, E. & Bryant, P. J. Adenosine deaminase-related growth factors stimulate cell proliferation in *Drosophila* by depleting extracellular adenosine. *Proc*. *Natl*. *Acad*. *Sci*. *USA* **99**, 4403-4408 (2002).

111. Radyuk, S. N. *et* *al*. Age-dependent changes in the transcription profile of long-lived *Drosophila* over-expressing glutamate cysteine ligase. *Mech*. *Ageing* *Dev*. **133**, 401-413 (2012).

112. Faisal, M. N. *et* *al*. Transcriptional regionalization of the fruit fly's airway epithelium. *PLoS* *ONE* **9**, e102534 (2014).

113. Strassburger, K. *et* *al*. Oxygenation and adenosine deaminase support growth and proliferation of *ex* *vivo* cultured *Drosophila* wing imaginal discs. *Development* **144**, 2529-2538 (2017).

114. Xu, C. *et* *al*. An *in* *vivo* RNAi screen uncovers the role of AdoR signaling and adenosine deaminase in controlling intestinal stem cell activity. *Proc*. *Natl*. *Acad*. *Sci*. *USA* **117**, 464-471 (2020).

**SUPPLEMENTAL FIGURES**


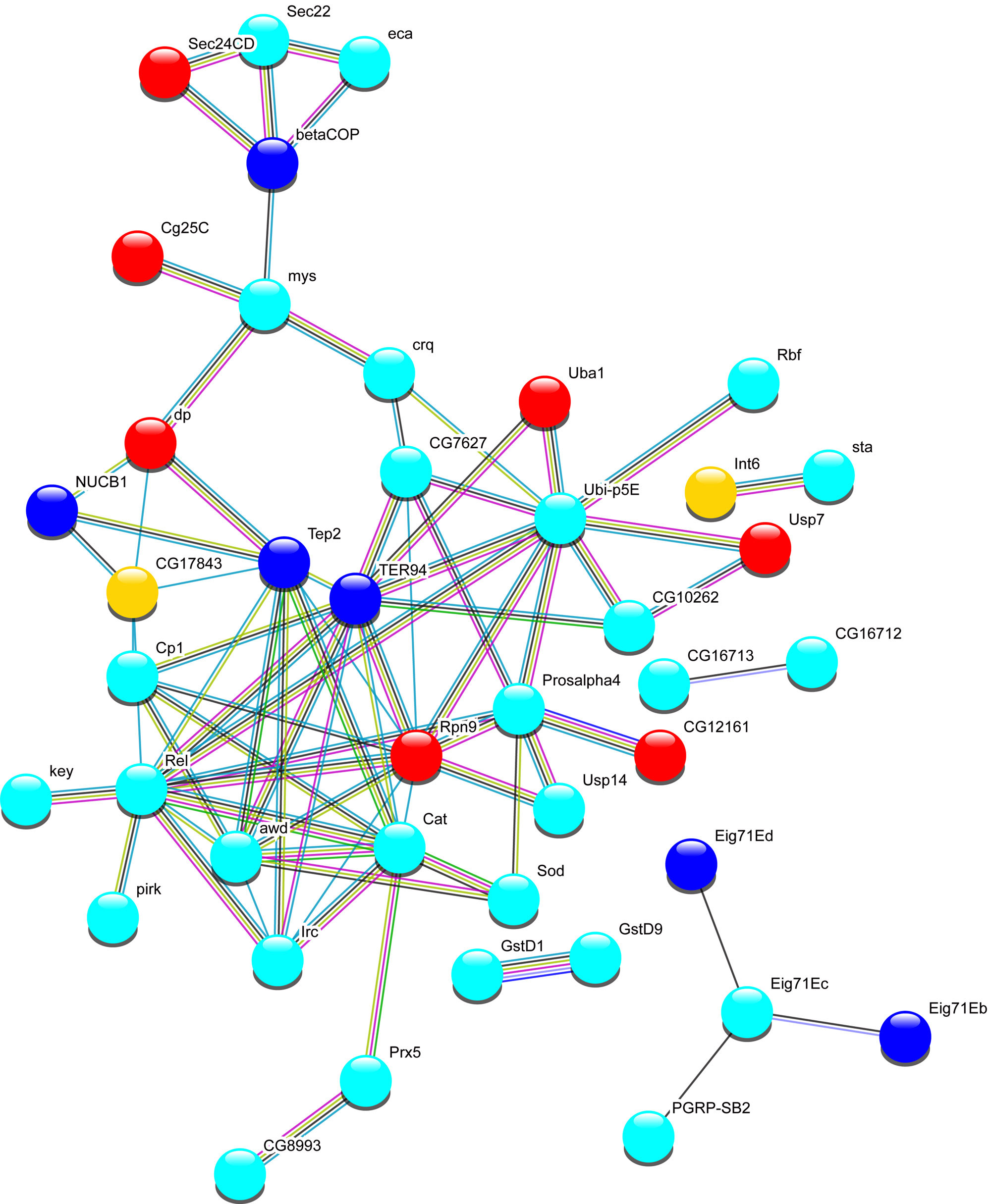


**Supplemental Figure 1**  The protein-protein functional interaction network predicted by STRING. The Search Tool for the Retrieval of Interacting Genes (STRING; [http://string-db.org](http://string-db.org/), vs 11.0)*^71^*,*^72^* was used to predict protein-interaction networks among the 125 proteins identified in the salivary gland apocrine secretion and exuvial fluid (Supplemental Table 1). The networks were obtained using 0.9 as the minimum required interaction score (highest confidence) and using the accession numbers tabulated in Supplemental Table 1. Since STRING does not distinguish between protein isoforms, the interactome was generated using 115 nodes. Support for an interaction is indicated by the edge color: known interactions from curated databases (blue-green) or from experimental determination (purple); predicted interactions from gene neighborhood (green), gene fusions (red), or gene co-occurrence (blue); and interactions based on textmining (light green), co-expression (black), or protein homology (bluish purple).

While individual proteins in the network are often known to be involved in multiple processes*^110^*^-^*^114^*, the central portion of the STRING interactome is occupied by many proteins that belong to different ontological categories and functional processes. The existence of multiple and strong protein-protein interactions suggests that the proteins in the apocrine secretion and exuvial fluid serve functional roles in processes critical to the metamorphosis from pupae to adult.

Connected nodes were recolored using Adobe Illustrator to indicate their previously demonstrated or hypothesized involvement in four such processes, conceptualized broadly: direct acting antimicrobial or antiviral defence (blue), systemic, upstream immune or antimicrobial, or stress response (cyan), moulting or lubrication (red), and homeostasis, metabolic, cellular or developmental processes (yellow). Evidence for these classifications is provided in the information accompanying Supplemental Table 1 and information from STRING provided in Supplemental Tables 2-4. For clarity, disconnected nodes are not shown. These are AIF, AttC, CecA1, CG9925, CG13551, CG14861, CG18607, Drsl6, Eig71Ea, Eig71Eg, Eig71Eh, Eig71Ej, Eig71Ek, MstProx, NimB2, os, and sick for direct acting antimicrobial/antiviral defense; Gale, Agdf0A, Atox1, cer CG1440, CG3328, CG3714, CG4580, CG6543, CG7924, CG9186, CG12384, CG13062, CG15111, CG16868, CG31704, CG34280, CG42713, CG42798, Cp1, Cys, Eip55E, ERp60, Fer2LCH, for, GS, ird1, l(2)37Cc, Manf, msl-3, Npc2a, Nplp2, Papss, polybromo, Spn77Ba, stck, Sur, Traf4, and Vps16B for systemic, upstream immune or antimicrobial, or stress response; CG1129, CG5897, CG8460, SppIII, Eno, Idgf3, Idgf4, jar, Mmp1, and Mur11Da for molting or lubrication; and capt, CG6656, CG6710, Lac, and neb for homeostatic, metabolic, cellular and/or developmental processes. The node of one protein, CG33998, has no known function and also is not shown.

**A** **B**


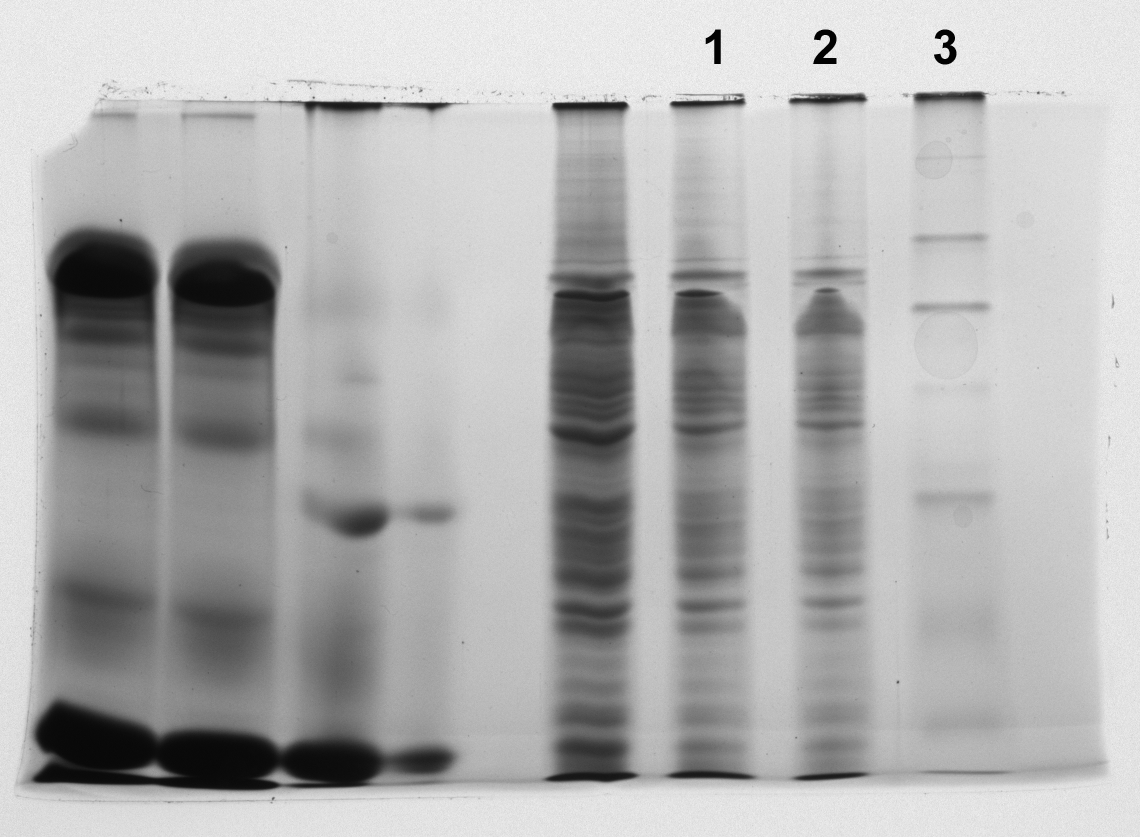

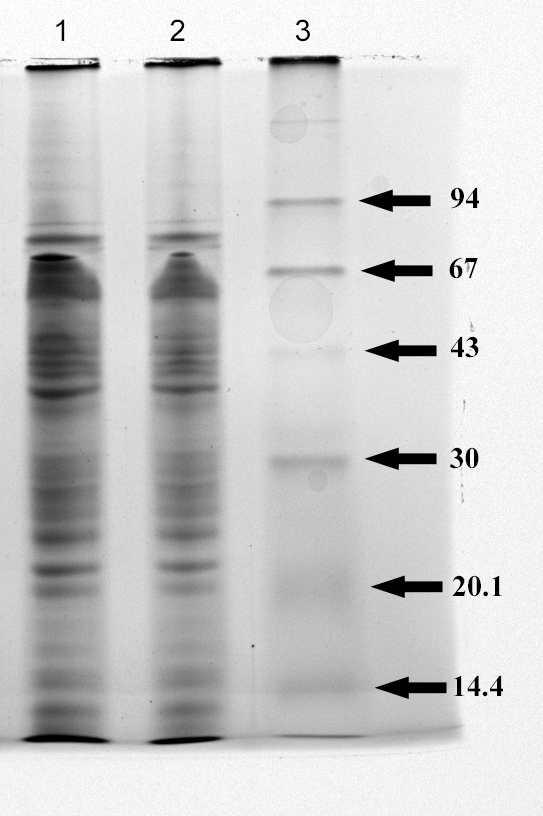


**Supplemental Figure 2** Panel **A** shows the original image of the SDS-PAGE gel used to separate the proteins extracted from apocrine secretion and exuvial fluids. Panel **B** shows a cropped image displaying only the lanes used in this analysis. Lanes 1-3 correspond to the same lanes in both panels. The unlabeled lanes in Panel A were not used in this analysis. Lane 1 = apocrine secretion from the prepupal salivary glands. Lane 2 = exuvial fluid. Lane 3 in panel B contains molecular weight standards as indicated by arrows. MW standards: 94 kDa phosphorylase B, 67 kDa bovine serum albumin, 43 kDa ovalbumin, 30 kDa carbonic anhydrase, 20.1 kDa trypsin inhibitor, 14.4 kDa a-lactalbumin (low MW calibration kit from Pharmacia/GE-Healthcare Corp.). SDS-PAGE was performed as described in the Materials & Methods. A lane the length of the 5.5 cm long × 8 cm wide slab gel (BioRad Protean Mini-II) was cut into 25 pieces and each was subject to mass spectrometric analysis.

**A**


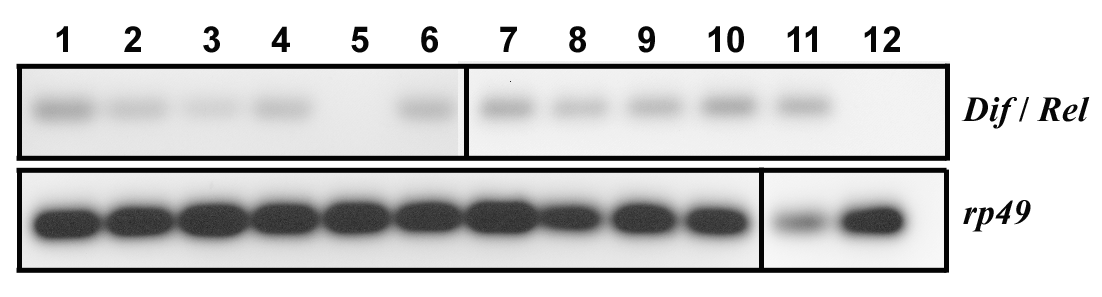


**B_1_**


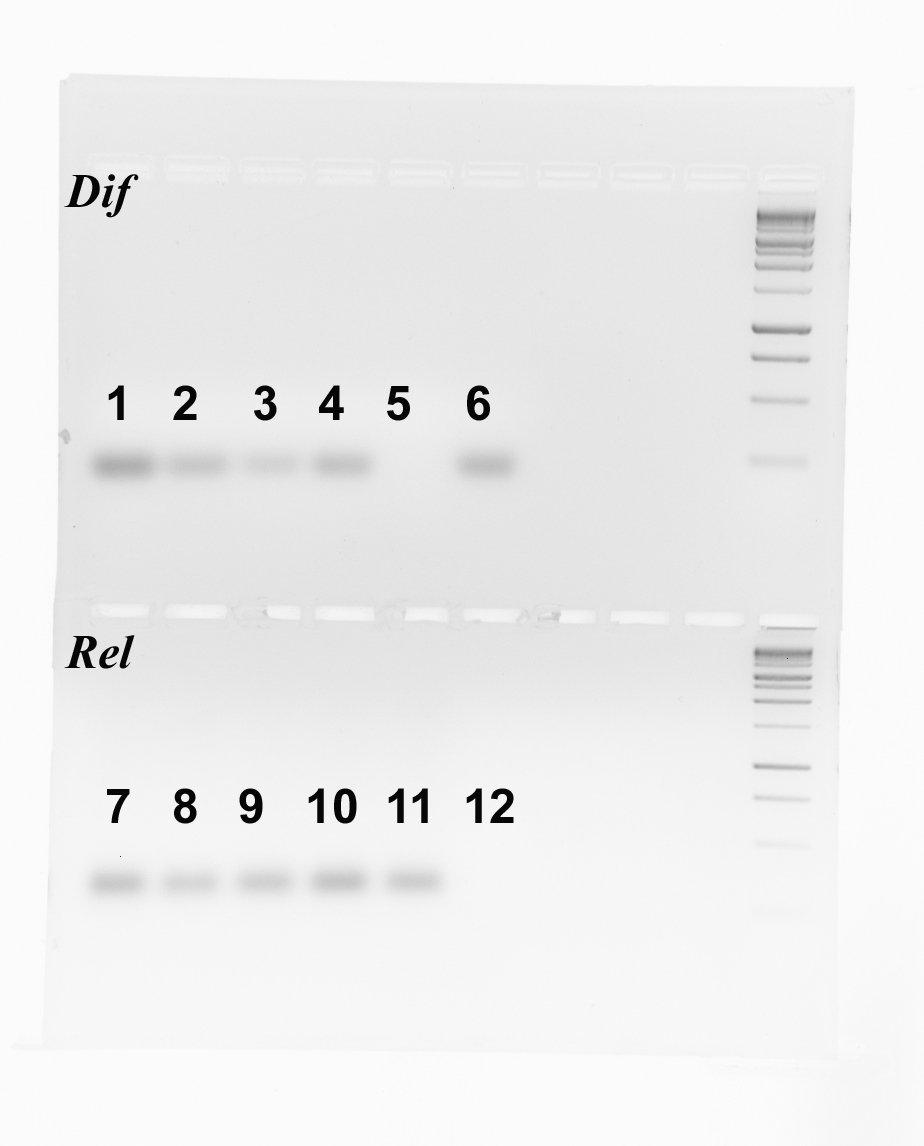


**B_2_**


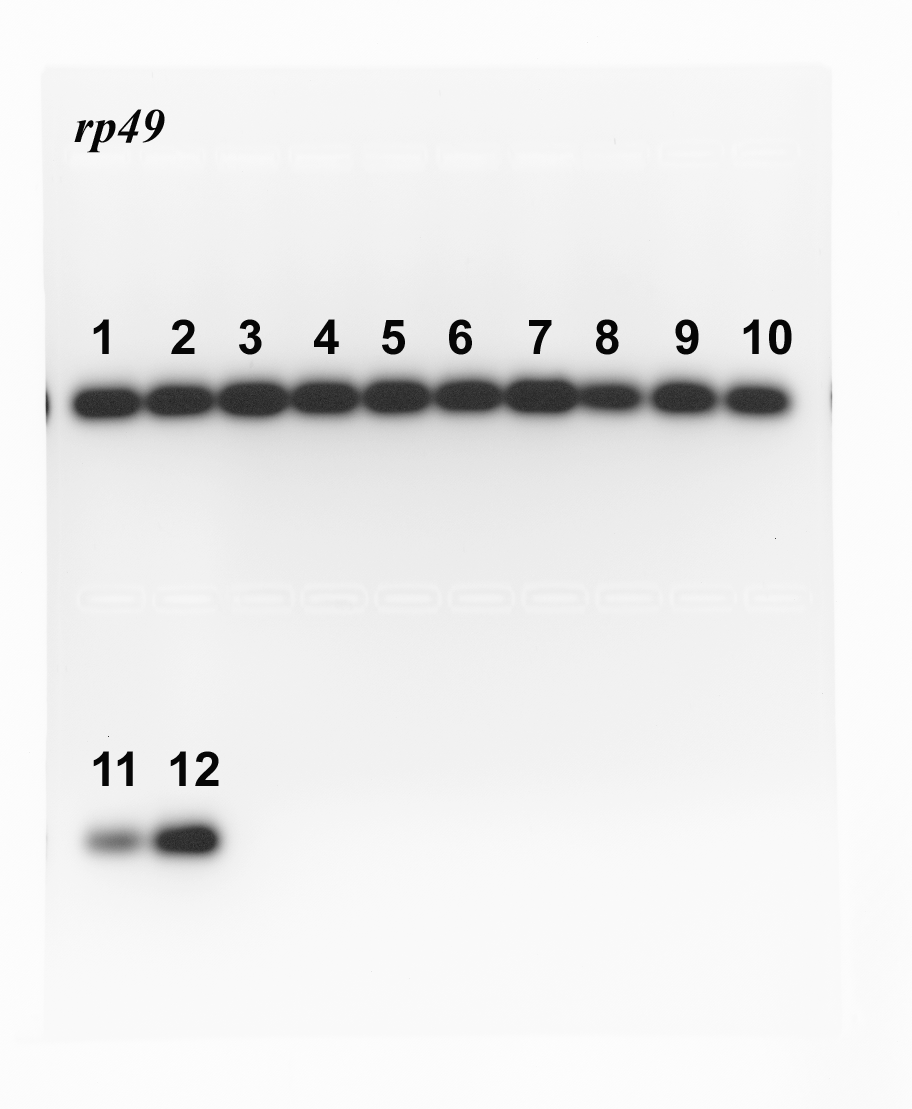


**Supplemental Figure 3**  Assessment of the effectiveness of RNAi knockdown by RT-PCR analysis of gene expression. Transcript levels were analyzed in poly(A)^+^ RNA samples extracted from SGs of individual genotypes at a unified time 5 hr APF. This provided sufficient time (minimum 23 hr) for the expression of *Gal4*-drivers and corresponded to a time point of 3 hr prior to apocrine secretion when the majority of proteins to be secreted into future exuvial fluid would have had to be synthesized. Standard RNAi conditions are expected to be sufficient to silence the function of a particular gene, and also prevent production of any protein, as even the tissue-specific expression of endogenous *Sgs4* gene and its related -*Gal4* driver start to decline few hours after pupariation.

The RT-PCR was performed as described previously*^30, 55^*. Briefly, RNA was extracted from 10 pairs of prepupal SGs in 10 μl 4 mol/L guanidium isothiocyanate, 1% SDS, 10 mmol/L Tris, pH 8.0, and the poly(A)^+^ RNA was isolated using an Oligotex suspension and mini-spin columns (Qiagen GmbH., Hilden, Germany) according to the manufacturer’s instructions. cDNA synthesis was performed in 10 μl from one-fifth of the extracted poly(A)^+^ RNA using the Moloney murine leukemia virus reverse transcriptase (Cetus-Perkin Elmer, Branchburg, NJ, USA) with reverse primers, followed by amplification with the AmpliTaq DNA polymerase (Cetus-Perkin Elmer) in the presence of forward and reverse primers. The *Dif* primers [reverse (5‘-CTTCAATGCCAAATTCGATCA-3’) and forward (5’-ATCGTGGAGGAGCCGACAAGC-3’] were selected from the common 3rd and 4th coding exons of the *Dif* gene (GenBank accession numbers L29015 and AC010121.7) so that between them is 105 nucleotides long intron. The expected RT-PCR product is 389 bp, whereas genomic DNA contamination in the sample (see also below) would give the detectably larger fragment of 494 bp. The *Rel* primers [reverse (5’- CTCTCCAGTTTGTGCCGACTT3’) and forward (5’-AAACCTTCCCGGAGGTTACAC-3’)] were selected from the common 1st and 2nd coding exons of the *Rel* gene (GenBank accession numbers U62005 and AC009258.3) so that between them is 105 nucleotides long intron. The expected RT-PCR product is 467 bp, whereas genomic DNA contamination in the sample (see also below) would give the detectably larger fragment of 755 bp. The *rp49* gene transcripts [primers: reverse (5’-GTGTATTCCGACCACGTTACA-3’) and forward (5’-TCCTACCAGCTTCAAGATGAC-3’)] were used as an control for whether approximately equal amounts of poly(A)^+^ RNA were used in the RT-PCR reactions.

The PCR cycles using a StratageneRobocycler 40, were as follows: denaturation 94°C for 1 min, primer annealing 54°C for 2 min, polymerase extension 72°C for 3 min, and the MgCl_2_ concentration was 1.5 mmol/L. The number of cycles was 30. To exclude DNA contamination, the extracted poly(A)^+^ RNA was treated with DNase devoid of RNase (Roche Diagnostics GmbH., Mannheim, Germany). RT-PCR products were separated by size on 2% agarose gels (9 cm wide × 11 cm long, OWL Easy Cast B1 apparatus with 10-well combs). To facilitate comparison of the differently sized *Dif* (389 bp) and *Rel* (467 bp) RT-PCR products, the *Rel* samples were initially loaded into alternate lanes, the gel system run for a short time (15-20 minutes) and then the *Dif* samples were loaded in the unused lanes. Multiple ten-lane gels were run in parallel to analyze the *Dif/Rel* RT-PCR products and the *rp49* RT-PCR products. The gels were then stained with ethidium-bromide and images obtained using a UVP Doc-It documentation system. The images were then cropped and assembled using Adobe Photoshop to produce the images in Panels A and B.

Panel **A**: Cropped negative images of the gel-resolved RT-PCR products. The upper row shows the levels of *Dif* and *Rel* RT-PCR products in the samples as numbered below (black vertical line between samples 6 and 7 in upper row and between samples 10 and 11 in lower row indicates the point of assembly from two independent gels shown in panels **B_1_** and **B_2_**):

1. *Dif* RT-PCR products in wild type *Oregon R* control animals

2. *Dif* RT-PCR products in *Sgs4-Gal4* animals

3. *Dif* RT-PCR products in *y^1^v^1^*; *P*{*TRiP*.*HM05191*}*attP2* (*UAS-Dif*^RNAi^*)* animals

4. *Dif* RT-PCR products in *y^1^v^1^*; *P*{*TRiP*.*HM05154*} *attP2* (*UAS-Rel*^RNAi^) animals

5. *Dif* RT-PCR products in *Sgs4-Gal4*>>*y^1^v^1^*; *P*{*TRiP*.*HM05191*}*attP2* (*UAS-Dif*^RNAi^*)* animals

6. *Dif* RT-PCR products in *Sgs4-Gal4*>>*y^1^v^1^*; *P*{*TRiP*.*HM05154*}*attP2* (*UAS-Rel*^RNAi^) animals

7. *Rel* RT-PCR products in wild type *Oregon R* control animals

8. *Rel* RT-PCR products in *Sgs4-Gal4* animals

9. *Rel* RT-PCR products in *y^1^v^1^*; *P*{*TRiP*.*HM05191*}*attP2* (*UAS-Dif*^RNAi^) animals

10. *Rel* RT-PCR products in *y^1^v^1^*; *P*{*TRiP*.*HM05154*} *attP2* (*UAS-Rel*^RNAi^) animals

11. *Rel* RT-PCR products in *Sgs4-Gal4*>>*y^1^v^1^*; *P*{*TRiP*.*HM05191*}*attP2* (*UAS-Dif*^RNAi^) animals

12. *Rel* RT-PCR products in *Sgs4-Gal4*>>*y^1^v^1^*; *P*{*TRiP*.*HM05154*}*attP2* (*UAS-Rel*^RNAi^) animals.

The lower row shows the profile of *rp49* gene expression in the same RNA sample used for the RT-PCR shown in the upper row.

Panels **B_1_** and **B_2_** show the original images of the agarose gels used to separate the RT-PCR products, that have been used to pick up, crop and assemble figure shown in Panel **A**.
